# Analysis of 17 cases of posterior vertebral column resection in treating thoracolumbar spinal tuberculous angular kyphosis

**DOI:** 10.1186/s13018-015-0195-7

**Published:** 2015-05-13

**Authors:** Tianhua Zhou, Chuan Li, Bin Liu, Xun Tang, Yongyue Su, Yongqing Xu

**Affiliations:** Department of Orthopedics, Kumming General Hospital of Chengdu Military Command, No.212 Daguan Road, Kunming, 650032 Yunnan China

**Keywords:** Spinal tuberculosis, Post-tuberculosis kyphosis, Posterior vertebral column resection, Osteotomy

## Abstract

**Objective:**

This study aims to explore the efficacy and safety of posterior vertebral column resection (PVCR) in treating thoracolumbar spinal tuberculous angular kyphosis (TSTAK).

**Methods:**

From January 2008 to January 2012, 17 TSTAK patients were treated surgically, including five males and 12 females, with an average age of 23.6 years, among five cases who had the kyphotic apical vertebrae located at the thoracic vertebrae, ten cases were located at the thoracolumbar segment, and two cases were located at the lumbar vertebrae. The kyphotic Cobb angle was measured in the preoperative, postoperative, and final follow-up, respectively, and the nerve function ASIA classification was assessed.

**Results:**

The mean operative time was 364 min; the average intraoperative blood loss was 2,218 ml; and the average intraoperative blood transfusion was 1,863 ml. Among the five patients with the preoperative nerve function as grade D, four of them recovered to grade E. The preoperative average Cobb angle was 81.3° ± 12.8°, while the postoperative average Cobb average was 17.3° ± 3.6°; while it was significantly improved than the preoperative (*P* < 0.01), the average kyphosis correction rate was 68.7% ± 6.5%; the postoperative average follow-up was 18.7 months, with an average correction loss as 3.3°.

**Conclusion:**

PVCR could be safely and effectively used in TSTAK.

## Introduction

According to the reports of WHO, no matter in the developed or developing countries, the occurrence of tuberculosis (TB) was showing a rising trend, and in 2011, more than 9 million new TB patients were reported around the world, as well as 1.4 million cases of death [1]. The rapid growth of global population was also one of the reasons for the continuous rise of TB cases. In the twenty-first century, mankind would continue to face the challenge of increased TB controlling [2]. About 20% of TB cases was the extrapulmonary tuberculosis [3], among which about 50% would involve the spine, and the spinal tuberculosis was more seen in the lower thoracic segment and the upper lumbar segment [4,5]. Most spinal tuberculosis patients could obtain the cure through a standardized anti-tuberculosis treatment, while most patients would remain to have some degree of kyphosis, and the kyphosis of 3% to 5% patients would exceed 60° [6,7]. The severe kyphosis would not only affect the patients’ appearance and interfere their mental status but also affect the trunk balance and cardiopulmonary functions and even cause the delayed neurological damages [8,9]. And the patients might often require the orthopedic surgeries due to the local persistent pain, dysfunctions, or cosmetic problems.

The correction surgery of thoracolumbar kyphosis should be based on the different kyphotic angles and the patients’ specific circumstances, thus using the anterior, posterior, or combined approach for the orthopedic surgeries. The anterior approach existed such defects as low kyphosis-orthopedic rate, often with residual kyphosis, postoperative fibrosis, and adhesions; and when the kyphosis was above 60° and the pleural adhesions existed, it would be difficult to expose the lesions [9,10]. Although the combined surgical approach exhibited satisfactory results, such shortcomings still existed as long surgical period, large trauma, and interference towards the cardiopulmonary functions, so it was also less used in the clinical practice [11,12]. In recent years, the spine surgeons had increasingly adopted the single posterior approach to complete the debridement of spinal tuberculosis, to correct the kyphosis, as well as to perform the inter-vertebral support-graft fusion and internal fixation [13-15], which can avoid entering the chest and impede bringing *Mycobacterium tuberculosis* into the chest. Currently, the posterior approaches towards the kyphosis surgery included the following: Smith-Petersen osteotomy (SPO), pedicle subtraction osteotomy (PSO), posterior vertebral column resection (PVCR), and multi-segmental improved vertebral column resection (MVCR), etc. From January 2008 to January 2012, in conjunction with our own clinical practice, our department applied PVCR to treat 17 thoracolumbar spinal tuberculous angular kyphosis (TSTAK) cases that had the Cobb angle >60° and reported as the following.

## Materials and methods

### General information

This study had 17 cases, including five males and 12 females; aged 9 to 41 years old, with the mean age of 23.6 years. This study was conducted in accordance with the declaration of Helsinki. This study was conducted with approval from the Ethics Committee of Kumming General Hospital. Written informed consent was obtained from all participants. The disease duration was 16 ~ 122 months, and the average duration was 37.5 months. Eight cases had the lesions involved in one to two vertebral bodies, and nine cases had the lesions involved in ≥3 vertebral bodies. And among the patients, five cases were post-thoracic tuberculosis (T5 ~ T10) kyphotic deformity, ten cases were post-thoracolumbar tuberculosis (T11 ~ L1) kyphotic deformity, and two cases were post-lumbar tuberculosis kyphotic deformity. The preoperative Cobb angles of all patients were >60°, among who the Cobb angles of 13 cases were 60° ~ 90°, and those of four cases were >90°, the biggest Cobb angle was 102°, and the average was 81.3° ± 12.8°. All patients could be seen with obvious kyphosis, while without such tubercular clinical symptoms as fever and night sweats, etc., the erythrocyte sedimentation rate and C-reactive protein were in the normal ranges. The preoperative nerve functional ASIA classification were the following: five cases were of grade D, 12 cases were of grade E (Table 1). The X-ray, and 2D and 3D CT imaging and MRI were performed preoperatively to make clear the location and extent of kyphosis, the involved vertebral numbers, as well as the compression and degeneration situations of vertebral spine.

**Table 1:** **Clinical data of patients**

**Case number**	**Age/gender**	**Follow-up (months)**	**Diseased segment**	**Kyphotic Cobb angle (°)**			**ASIA**	
				**Preop**	**Postop**	**Final follow-up**	**Preop**	**Final follow-up**
1	22/M	14	T4–T7	74	15	19	D	D
2	41/M	13	T5–T6	60	16	20	D	E
3	28/M	17	T5–T6	64	15	18	E	E
4	25/W	16	T5–T9	86	18	21	D	E
5	24/W	15	T7–T9	76	18	21	E	E
6	9/W	27	T11–L1	72	15	18	D	E
7	16/W	15	T11–T12	66	10	12	E	E
8	29/M	17	T11–T12	76	16	19	E	E
9	28/W	22	T11–T12	96	19	23	E	E
10	30/M	15	T11–L1	74	17	21	E	E
11	23/W	23	T12–L1	82	19	22	E	E
12	26/W	20	T12–L1	88	17	20	E	E
13	14/W	17	T12–L2	92	17	19	E	E
14	15/W	20	T12–L2	100	22	25	E	E
15	28/W	16	T12–L2	86	16	21	E	E
16	16/W	34	L1–L3	102	28	32	D	E
17	28/W	16	L2–L3	88	16	19	E	E

### Surgical procedures

All the patients were performed with the tracheal intubation for the general anesthesia, then pronely laid on the bow rack, the special attention should be paid to prevent neck hyperextension, in which situation the eyes and abdomen would be uncompressed; then set the convex apical vertebrae as the center, extended three to four vertebrae upwards and downward, and performed the median incision along the spinous process. The dermal, subcutaneous and lumbodorsal fascia were incised by turns, the paraspinal muscles was stripped from the subperiosteum to fully reveal the vertebral laminas, facet processes, transverse process, and partial ribs, and the surgical field must be spacious enough to ensure that the osteotomic site could be performed the periosteum-adhesive separation in the front of transverse process. The C-arm X-ray machine was used for the apical vertebral positioning then placed the bilateral pedicle screws in superior and inferior two to three vertebrae of PVCR vertebra. According to the preoperative planning and referring of the intraoperative lateral imaging results, the osteotomic border was marked, and the spinous process, vertebral laminas, facet processes, and transverse processes were resected according to the marks; the thoracic vertebra also needed to be cut 3 ~ 5 cm rib, costal diapophysis and rib head; the opposite side was fixed with the temporary fixation bars to ensure the spinal stability before the spinal anterior column osteotomy, thus the possible spinal cord injury might be avoided (if the osteotomy was performed in the thoracic vertebrae, 500-mg methylprednisolone injection should be intravenously dropped to protect the spinal cord). The vertebral pedicle and the outlet nerve root, which was in the lower edge of vertebral arch pedicle, was revealed and carefully freed 3 ~ 5 cm outwards along the nerve root, so that the nerve roots could be gently pull aside (if it was the thoracic nerve root, it could be ligated and cut 3 cm outside from the intervertebral foramen) then tightly stuck to the subperiosteum of vertebral lateral side. The nerve roots were stripped and pushed until the anterior longitudinal ligament, the vertebral sledge plate was used to protect such important organs as the anterior vertebral great vessel, vertebra-segmental vessels, and parietal pleura, and the compression hemostasis was also performed. Under the direct vision and careful protection of spinal cord, the vertebral body and intervertebral disc on one side were gently removed as planned by the bone knives, nucleus pulposus pliers, and curettes. When it was completed, the temporary fixation bar was exchanged, and the contralateral vertebral body was resected with the same method. The entire spine removal was completed when the bilateral resections met, then the sneak expansion was performed when decompressing the adjacent vertebral lamina. The distraction forceps were used to maintain an appropriate distraction in the anterior vertebral bodies, while the rear bars were replaced alternatively and with the appropriate pressure to gradually correct the kyphosis. The residual defect heights of anterior vertebral bodies were measured and filled with the titanium cage, which were mixed with streptomycin powder and small autologous bone pieces and had the suitable diameter (for the thoracic vertebra, 1.6 cm; for the lumbar vertebra 1.9 cm) and high. Thus, the intervertebral-supporting vertebra fusion was performed; the autologous bone pieces were implanted and pressed tightly into the both sides of titanium cage then replaced the preflex fixation rods, so that the rear pressure-fixation was performed to complete the deformity orthopedics. The orthopedic procedure must be carried out slowly to observe whether the spinal cord exhibited the folds and compression. At the same time, the loosening or unplugging of the pedicle screws should be noticed. Once the orthopedic surgery is completed, the wake-up test should be performed timely to prevent the occurrence of paraplegia. After that, the posterior lateral and intervertebral graft fusion were performed, the drainage tubes were placed, and the wound was closed.

This study resected three cases of single residual lesion vertebra, five cases of two residual lesion vertebrae, and nine cases of three residual lesion vertebrae. The average resection of residual lesion vertebrae was 2.35. The fixed segments were generally two segments upper and lower in the lumbar segments, three segments upper and lower in the thoracic segments, and upper three segments and lower two segments in the thoracolumbar segments, respectively. All the patients exhibited no significant sagittal imbalance before and after the surgery.

### Postoperative treatment

After the surgery, such conventional treatments as antibiotics, phlegm-resolving, and necessary supportive treatments were performed. Meanwhile, the patients’ vital signs, movements of lower limbs, and feeling of the perineal region were closely observed, and all kinds of situations should be timely resolved. The patients with thoracic segment osteotomy were routinely given 500-mg methylprednisolone injection on the same postoperative day to reduce the possible spinal cord edema. The drainage tubes were withdrawn 48 ~ 72 h later. The patients were asked to be in strict bed confinement for 3 ~ 6 weeks while performing the simultaneous rehabilitation therapies such as ankle joints’ flexion and extension, lower limbs’ alternating raising and kicking, etc., as well as urinary catheter occlusion and massage. After that, the patients could wear the customized wearable brace for ambulation.

### Postoperative follow-up and efficacy evaluation

All the patients undergone the normal and lateral lumbar X-ray examinations on the 3rd, 6th, 12th, and 24th month to understand the situations of orthopedic correction, internal fixation, and graft fusion, their Cobb angle were measured, and their 2D and 3D CT imaging were reviewed when necessary. The pre- and postoperative nerve functional recoveries were assessed by ASIA classification standards.

### Statistical analysis

The SPSS 12.0 statistical software was used, the measurement data were expressed as $\overline{x}\pm s$, and the comparison of the Cobb angles, before and after the surgery, used the paired *t* test, with *P* < 0.05 considered as the statistical significance.

## Results

**Figure 1:**
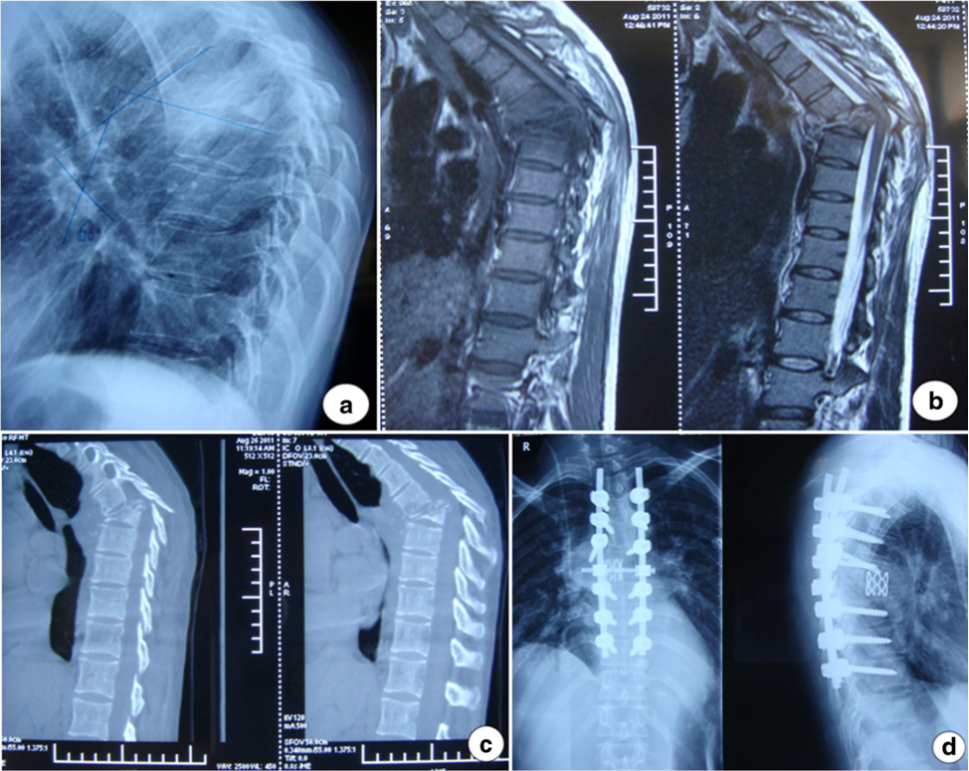
A 41-year-old woman who presented with 60° post-tubercular angular kyphosis in the 5th and 6th thoracic vertebrae. Preoperation X-ray **(a)**, MRI **(b)**, CT images **(c)**. After PVCR procedure, her kyphosis were corrected to 16° **(d)**.

**Figure 2:**
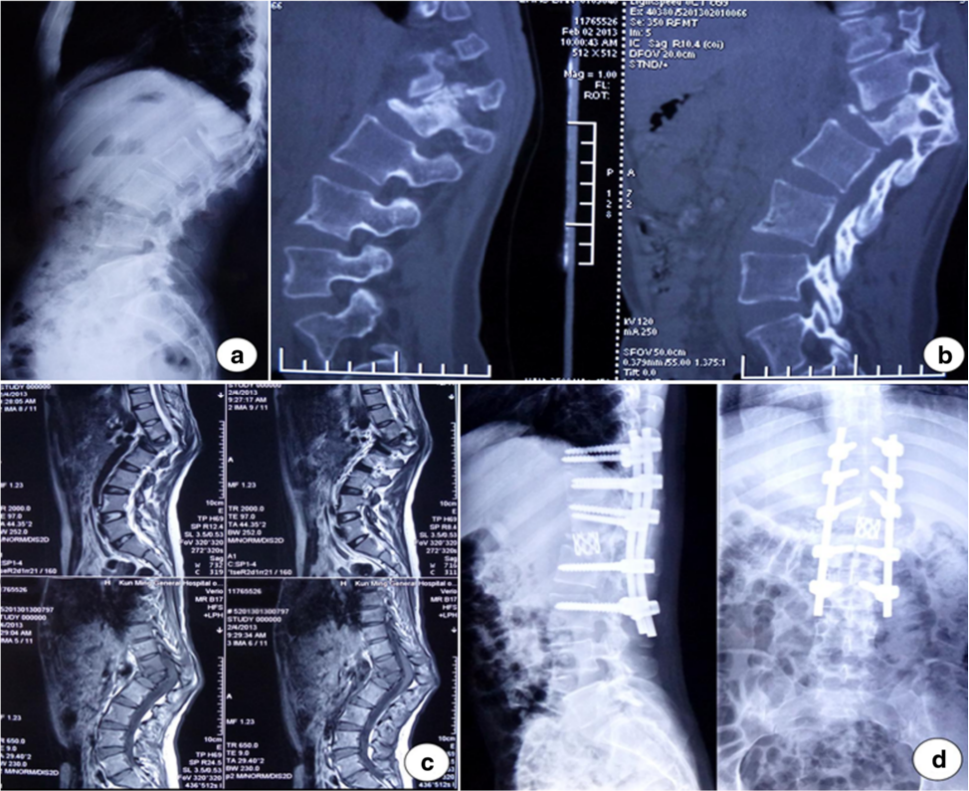
A 23-year-old woman who presented with 82° post-tubercular angular kyphosis in the 12th thoracic vertebrae and 1st lumbar vertebrae. Preoperation X-ray **(a)**, CT images **(b)**, MRI **(c)**. After PVCR procedure, her kyphosis were corrected to 19° **(d)**.

The operative time was 330 ~ 450 min, with the average as 364 min; the intraoperative blood loss was 1,600 ~ 2,500 ml, with the average as 2,218 ml; and the intraoperative blood transfusion was 1,000 ~ 2,000 ml, with the average as 1,863 ml. During the surgery, two patients had pleural rupture, while timely patched, and no pneumothorax or blood pneumothorax was observed. All cases exhibited postoperative first-stage healing, without the sinus formation, internal fixation loosening, or nerve function aggravation. Five patients were in grade D of nerve functions preoperatively, while four of them returned to grade E after the surgery. The preoperative Cobb angle was 60° ~ 102°, with an average of 81.3° ± 12.8°. The postoperative Cobb angle was 10° ~ 28°, with an average of 17.3° ± 3.6°, which was significantly improved than the preoperative (*P* < 0.01). The average kyphotic correction rate was 68.7% ± 6.5%; the postoperative follow-up was 13 to 34 months, with an average of 18.7 months. In the final follow-up, the Cobb angle was 10° ~ 32°, with an average of 20.6° ± 3.9°, which lost an average of 3.3° than the postoperative (*P* > 0.05) but still significantly improved than the preoperative (*P* < 0.01). The typical cases were shown in Figures 1 and 2.

## Discussion

Such a variety of diseases as the spinal tuberculosis, congenital spinal deformity, and spinal trauma could lead to the increase of spinal kyphotic angle, which were the common causes of kyphosis. Among them, the spinal tuberculosis would often destroy the anterior and middle vertebral spine and damage the growth plates in children, while the posterior spine would continue to grow. Thus, the kyphosis would often be resulted in, and it was the most common cause of kyphosis. When the kyphosis was greater than 90°, it might induce the delayed neurological damages, even the paralysis. Thus, it usually required the surgical orthopedic treatment [16,17].

The surgical methods towards the kyphosis included SPO, PSO, VCR, and MVCR, etc. The angle that could be corrected by the single segment SPO was limited, typically 10° ~ 20° [18], while that of single segment PSO was improved to 30° ~ 40° [19]. VCR had the strongest correction ability towards the kyphosis, reaching up to 49° ~ 80° [16,17,20]. From the results of these literatures, we could see that SPO and PSO were only suitable for mild to moderate kyphosis while not satisfied towards the severe kyphosis; VCR could gain the satisfactory results towards the severe kyphosis, while it could not be used towards the mild to moderate kyphosis, because the skill difficulties of VCR were bigger, with bigger trauma and higher risk. In this study, the kyphotic angles of all patients were >60°. Thus, the PVCR technology was applied, which could really shorten the operation time, reduce the trauma, and the effects were not worse than the combined anterior-posterior approach VCR, the results showed that the preoperative average Cobb angle was 81.3°, while the postoperative average Cobb angle was 17.3°. The average kyphosis correction rate was 68.7%, and the average kyphosis correction angle was 64°. So, the orthopedic results were satisfactory. We thought that the PVCR technology was the effective method for the correction of severe kyphosis, but because the technical difficulties were huge, the risks were high. It must be cautiously and gradually carried out by the highly qualified spine surgeons. Otherwise, the catastrophic complications might appear.

Since Suk et al. [21] reported PVCR in 2002, this technology had been more and more widely used in all areas such the spinal deformity, severe trauma, and tumors but less in spinal tuberculosis. The PVCR technology simplified the anterior-posterior combined-approach VCR technology. Through the single posterior approach, it could complete the resection of posterior, middle, and anterior vertebral column, the 360° nerve decompression surrounding the dural sac, and the inter-vertebral body graft fusion. It reduced the case selection that needed the combined anterior-posterior approach previously. Thus, it was a major advance in spinal surgical technologies. Compared with the VCR technology, the PVCR technology could not only reduce the surgical trauma and shorten the operation time but also avoid thoracotomy for the patients with thoracic lesions, reduce interference of the patients’ cardiopulmonary functions, and increase the surgical safety. Based on these advantages, we had gradually applied the PVCR technology into the clinical practice of partial severe spinal trauma, cancer, and tuberculosis and achieved the satisfactory results.

The PVCR technology required the dural sac-surrounding 360° nerve decompression, spinal osteotomy, decompression, and fixation to be performed at the edge of spinal cord, which required the precise surgical procedures, soft and proper intraoperative usage of temporary fixation rods, so any slight intraoperative accidence might cause the neurological damage, even paralysis. Among the 70 cases reported by Suk et al. [21], there were two cases with complete spinal cord injury. Dorward and Lenke [22] reviewed and reported that the average neurological complication rate was up to 14.3%. This showed that PVCR was still the surgery with high risks and difficulties. Therefore, we must master the basic SPO, PSO, and other spine osteotomy technologies then strictly judge the indications, gradually and carefully carry out this surgery, and should never blindly speed up. Only in this way could such catastrophic consequence of the spinal cord be avoided.

Starting from the surgical positioning, we should carefully check all the details, such as that the abdomen should be vacant, the bony prominence should be padded softly, the eyes could not be under pressure, and the neck could not be in the hyperextension, etc. The surgical revealing must be full for the operation under the direct vision. The revealing of vertebral body must be close to the subperiosteum, so that the damages to such adjacent vital structures as the vertebral segmental vessels and thoracoabdominal large vessels could be avoided. When performing the vertebral osteotomy, revealing must be full, the operation must be under the direct vision, the bone knife must be sharp to avoid excessive vibrations, and the dural sac should not be interfered as much as possible. The temporary fixation rods should be placed on the contralateral side to ensure no spinal slippage during the spinal osteotomy. The time of correcting the kyphosis was the time that the neurological complications were most prone to occur. Lenke et al. [23] reported that about 50% spinal cord injuries occurred at this time; the laminar decompression and the moderate sneak expansion of two adjacent faces should be completed in advance, meanwhile moderately distracting the vertebral osteotomic planes, to avoid excessive crispation, accumulation, and iatrogenic spinal obstruction when the posterior spinal columns closed; the closure of the posterior spinal column should be gradual and steady, completed fractionally. Once the deformity correction was completed, the intraoperative wake-up test should be performed to understand the situations of spinal functions; if the lower limbs were found unmovable, the degree of deformity correction should be significantly reduced while rapidly infused with 1.0-g methylprednisolone to rescue the spinal functions. When the intervertebral titanium cage-supporting graft was completed, both sides of titanium cage should be moderately implanted with the autologous bone pieces and pressed with fingers to increase the bone graft effect; as for the cases that had the gap <3 mm after the laminar closure, the vertebral graft fusion could be simultaneously performed.

This study had no intraoperative and postoperative complications, which was related with the small case numbers and not-long-enough follow-up period, thus there existed a certain degree of limitation. The analytic results might thus exist certain statistical deviation. But in general, PVCR was safe and effective in treating TSTAK, as long as the preparations were proper and full. It could effectively improve the kyphosis correction rate while not increasing the perioperative complications.

## References

[1] WHO: 2008 Tuberculosis facts. 2008. http://www.who.int/tb/publications/2008/factsheet_april08.pdf. Accessed 21 March 2013.

[2] Lawn SD, Zumla AI (2011). Tuberculosis. Lancet.

[3] Ozvaran MK, Baran R, Tor M, Dilek I, Demiryontar D, Arinç S (2007). Extrapulmonary tuberculosis in non-human immunodeficiency virus-infected adults in an endemic region. Ann Thorac Med.

[4] Jain AK (2010). Tuberculosis of the spine: a fresh look at an old disease. J Bone Joint Surg Br.

[5] Sun L, Song Y, Liu L, Gong Q, Zhou C (2013). One-stage posterior surgical treatment for lumbosacral tuberculosis with major vertebral body loss and kyphosis. Orthopedics.

[6] Moon MS (1997). Tuberculosis of the spine, controversies and a new challenge. Spine.

[7] Issack PS, Boachie-Adjei O (2012). Surgical correction of kyphotic deformity in spinal tuberculosis. Int Orthop.

[8] Tuli SM (1995). Severe kyphotic deformity in tuberculosis of the spine. Int Orthop.

[9] Jain AK, Dhammi IK, Jain S, Mishra P (2010). Kyphosis in spinal tuberculosis—prevention and correction. Indian J Orthop.

[10] Rajasekaran S, Prasad Shetty A, Dheenadhayalan J, Shashidhar Reddy J, Naresh-Babu J, Kishen T (2006). Morphological changes during growth in healed childhood spinal tuberculosis: a 15-year prospective study of 61 children treated with ambulatory chemotherapy. J Pediatr Orthop.

[11] Moon MS, Woo YK, Lee KS, Ha KY, Kim SS, Sun DH (1995). Posterior instrumentation and anterior interbody fusion for tuberculous kyphosis of dorsal and lumbar spines. Spine.

[12] Wang Z, Yuan H, Geng G, Shi J, Jin W (2012). Posterior mono-segmental fixation, combined with anterior debridement and strut graft, for treatment of the mono-segmental lumbar spine tuberculosis. Int Orthop.

[13] Zhang HQ, Wang YX, Guo CF, Zhao D, Deng A, Wu JH (2011). One-stage posterior focus debridement, fusion and instrumentation in the surgical treatment of cervicothoracic spinal tuberculosis with kyphosis in children: a preliminary report. Childs Nerv Syst.

[14] Djientcheu VP, Mouafo Tambo FF, Ndougsa IS, Ndougsa IS, Eloundou NJ, Kouna Tsala IN (2013). The role of surgery in the management of Pott’s disease in Yaoundé. A review of 43 cases. Orthop Traumatol Surg Res.

[15] Zhang H, Sheng B, Tang M, Guo C, Liu S, Huang S (2013). One-stage surgical treatment for upper thoracic spinal tuberculosis by internal fixation, debridement, and combined interbody and posterior fusion via posterior-only approach. Eur Spine J.

[16] Rajasekaran S, Vijay K, Shetty AP (2010). Single-stage closing-opening wedge osteotomy of spine to correct severe post-tubercular kyphotic deformities of the spine: a 3-year follow-up of 17 patients. Eur Spine J.

[17] Wang Y, Zhang Y, Zhang X, Huang P, Xiao S, Wang Z (2008). A single posterior approach for multilevel modified vertebral column resection in adults with severe rigid congenital kyphoscoliosis: a retrospective study of 13 cases. Eur Spine J.

[18] Burton DC (2006). Smith-Petersen osteotomy of the spine. Instr Course Lect.

[19] Kim KT, Park KJ, Lee JH (2009). Osteotomy of the spine to correct the spinal deformity. Asian Spine J.

[20] Lenke LG, Sides BA, Koester LA, Hensley M, Blanke KM (2010). Vertebral column resection for the treatment of severe spinal deformity. Clin Orthop Relat Res.

[21] Suk SI, Kim JH, Kim WJ, Lee SM, Chung ER, Nah KH (2002). Posterior vertebral column resection for severe spinal deformities. Spine.

[22] Dorward IG, Lenke LG (2010). Osteotomies in the posterior-only treatment of complex adult spinal deformity: a comparative review. Neurosurg Focus.

[23] Lenke LG, O’Leary PT, Bridwell KH, Sides BA, Koester LA, Blanke KM (2009). Posterior vertebral column resection for severe pediatric deformity: minimum two-year follow-up of thirty-five consecutive patients. Spine.

